# Organ-on-chip models: new opportunities for biomedical research

**DOI:** 10.4155/fsoa-2016-0038

**Published:** 2016-07-06

**Authors:** Alexander S. Mosig

**Affiliations:** 1 Center for Sepsis Control & Care, Jena University Hospital, Jena, Germany; 2 Institute of Biochemistry II, Jena University Hospital, Jena, Germany

**Keywords:** animal model, bioanalysis, biotechnology, organ-on-chip, translational research

Animal models are frequently used in biomedical research. However, recently a controversial debate about the transferability of data obtained in mouse models to human conditions emerged. Although cell-based *in vitro* approaches can be an alternative, conventional cell culture methods hardly reflect cellular cross-communication and neglect essential physiological parameters. Biochip-embedded tissue culture allows an optimal supply with nutrients and oxygen, an efficient removal of catabolic metabolites, and enables a physiological cell polarization and communication within tissues creating a complex physiological microenvironment *in vitro*. The combination of animal as well as human organ-on-chip with reliable *in vivo* models will create new possibilities for an integrative research strategy covering analysis from the cellular mechanistic level *in vitro* to the phenotype level *in vivo*.

Standard 2D cell culture still represents the most widely used technique to study cellular behavior, for example, in response to altered signaling pathways or treatment with pharmacological substances. However, its relative ease of use frequently is accompanied by severe limitations. Enrichment of waste products, limited supply with nutrients restricted by slow diffusion or lack of cell type-specific mechanostimulatory forces often result in rapid dedifferentiation of cells under these nonphysiological conditions. Novel microfluidically perfused organ-on-chip technologies represent innovative alternatives to circumvent these problems, allowing the establishment of organ models mimicking the 3D arrangements of cell types and cell layers close to physiological conditions [1].

Recently developed organ-on-chip systems are composed of multiple organ-specific cell types that are arranged in a bioinspired fashion resembling key features of organ-specific morphology. Although the research field of organ-on-chip models is still in its infancy, some of these models are already capable of closely reflecting *in vivo* cell–cell communication thereby creating an endogenous microenvironment that resembles key issues of organ physiology with defined organ-specific functions *in vitro*. Bhatia and Ingber [2] recently defined organs-on-chip as “…microfluidic devices for culturing living cells in continuously perfused, micrometer-sized chambers in order to model physiological functions of tissues and organs”. Organ-on-chip models in microfluidically supported biochips offer the possibility to more precisely regulate environmental conditions critical for growth of individual cell types. Miniaturized biochips allow the control of nutrition supply and removal of cellular waste products or secondary metabolites accumulating within the culture medium. Furthermore, oxygenation levels, hydrostatic pressure or shear stress can be can be adjusted to control for example maintenance of barrier integrity of cell layers and the control of cell migration *in vitro*.

Most organ-on-chip models include porous membranes as cell substrate around which defined cell layers can be arranged. These membranes create an interface that allows cellular crosstalk of, in other words, endothelial and epithelial cell layers [3]. Several groups have established organ models of the kidney [4,5], intestine [6,7], lung [8,9], heart [10] or the blood–brain barrier [11,12] based on this concept. In a 3D model of the human liver it has been shown that the co-culture of different organ-specific cell types (hepatocytes with endothelial cells, macrophages and stellate cells) creates a microenvironment that stabilizes individual differentiation and functionality of individual cell types [3]. A unilateral perfusion protocol triggers alignment and polarization of hepatocytes, thereby prohibiting dedifferentiation as a prerequisite for preserved long-term function *in vitro* [3,13]. Biochips also allow the integration of circulating immune cells and monitoring of their interaction under flow conditions with the biochip-embedded tissue. In this context it was recently demonstrated that the adhesion and migration of circulating monocytes is able to trigger tissue repair and repolarization of tissue-resident macrophages within a liver-on-chip model. The cellular signaling events occurring under these conditions were found to be associated with the recovery of hepatocellular functionality even in the presence of a persisting inflammation [14]. Similarly, in other organ-on-chip models it has been reported that compared with conventional maintenance in 2D culture systems, 3D cell culture offers improved conditions for establishment of stable cell–cell contacts mediating, for example, polarized growth and in consequence signal exchange that enhance long-term preservation of cell integrity [15,16].

To ensure a high transferability of results obtained with organ-on a chip models to the human *in vivo* situation, the assembly of organ models with cells of human origin is desirable. Here, the use of either primary cells or established cell lines has to be considered. Either one has its advantages and disadvantages. Primary cells usually show no signs of substantial cellular dedifferentiation or genetic alterations upon isolation and at first sight appear best suited for the intended use. However, at least in conventional cell culture they often tend to dedifferentiate during the further propagation or subculture. Although this phenomenon can be reduced under microfluidically supported growth conditions, propagation of primary cells in most cases is only possible for a limited number of passages. Therefore, constant monitoring of the cell differentiation state is necessary. In addition, it has to be considered that primary cells in most cases are isolated from patients that have undergone organ tissue resections alongside therapy and thus often have received extensive medication, for example, with cytostatic drugs ahead of surgical intervention. This medication-related bias may further contribute to an increased variability in the obtained results.

By contrast, immortalized cell lines show a relatively low variability compared with the primary cells. However, depending on the individual cell line they show various signs of cellular dedifferentiation and significant differences in function compared with the freshly isolated primary cells. Moreover, for generation of genetically modified cells using RNAi or CRISPR technologies, cell lines appear as an alternative. Their integration into biochips represents an efficient way to address the specific function of individual genes or proteins within an environment more closely resembling the *in vivo* situation. Human induced pluripotent stem cells (iPSCs) represent another alternative even when generated from patients with a particular disease. They have the ability of indefinitely self-renew and can be differentiated into virtually any cell type. However, the differentiation into the correct cell type within the organ-on-chip model needs to be tightly controlled to ensure reliable results. In this context it is noteworthy that it was recently shown that an improved generation of iPSCs by cell transfection with modified mRNA can be performed in a favorable microenvironment already created within a microfluidically supported biochip [17].

In addition to humanized models, the use of animal organ-on-chip models is also an interesting option. In particular, the combination of animal and human organ-on-chip models assembled in a similar manner and used under identical experimental conditions allows the identification of interspecies-related differences as well as common regulatory signaling pathways. An integrative research strategy of *in vitro* and *in vivo* models allows mechanistic studies at the cellular level within biochips as wells as systemic analysis within genetically and species-matched animal models. With regards to the recently emerged controversial debate about the transferability of data obtained in animal models to human conditions [18,19], such an integrative research strategy can be an alternative to conventional cell culture methods and animal experimentation. Organ-on-chip models are hereby not necessarily cheaper than animal models, but add various new interesting options to biomedical research. Considering the rapidly evolving technological possibilities of microsystem/microfluidic technology on one hand and the increasing knowledge in tissue engineering and regenerative medicine on the other hand, future organ-on-chip developments will certainly provide exciting new research and screening tools for basic research and drug screening.
